# A phase I study of neoadjuvant chemotherapy with gemcitabine plus oral S-1 for resectable pancreatic cancer

**DOI:** 10.3892/mco.2013.133

**Published:** 2013-05-27

**Authors:** HIDEHIRO TAJIMA, HIROHISA KITAGAWA, TOMOYA TSUKADA, SHINICH NAKANUMA, KOICHI OKAMOTO, SEISHO SAKAI, ISAMU MAKINO, HIROYUKI FURUKAWA, KEISHI NAKAMURA, HIRONORI HAYASHI, KATSUNOBU OYAMA, MASAFUMI INOKUCHI, HISATOSHI NAKAGAWARA, TOMOHARU MIYASHITA, HIDETO FUJITA, HIROSHI ITOH, HIROYUKI TAKAMURA, ITASU NINOMIYA, SACHIO FUSHIDA, TAKASHI FUJIMURA, TETSUO OHTA

**Affiliations:** Department of Gastroenterologic Surgery, Division of Cancer Medicine, Graduate School of Medical Science, Kanazawa University, Ishikawa 920-8641, Japan

**Keywords:** pancreatic cancer, gemcitabine, S-1, neoadjuvant chemotherapy, phase I

## Abstract

The aim of this study was to determine the maximum-tolerated dose (MTD), the dose-limiting toxicity (DLT) and the recommended dose (RD) of neoadjuvant chemotherapy (NAC) with gemcitabine (GEM) plus oral S-1 in patients with resectable pancreatic cancer. Thirteen patients with radiologically proven resectable pancreatic cancer were included in this study. S-1 was administered orally for 14 consecutive days, and GEM was administered on days 8 and 15 for two pre-operative cycles. The dose of S-1 in this study was planned with fixed doses of GEM (1,000 mg/m^2^): 20, 30 and 40 mg/day for levels 0, 1 and 2, respectively. Treatment was initiated at level 1 in 3 patients, while adverse events occurred in 2 patients during the second course, leading to a dose reduction to level 0 for the 8 remaining patients. Two of the 10 patients enrolled at level 0 were excluded. Of the remaining 8 patients, GEM administration was terminated due to DLT on day 15, during the first course in 3 patients, while level 0 dosage reached MTD. Surgery was performed for the remaining 11 patients included in the study. Post-operative complications included pancreatic fistulas in 5 patients and *Pseudomonas aeruginosa* sepsis in 1 patient. Two of the 11 patients exhibited a partial response and 9 patients stable disease. Eight of the 11 tumor specimens showed histopathological evidence of tumor cell injury. In conclusion, NAC with GEM and S-1 was not well-tolerated in this study. However, pre-operative chemotherapy may be effective against pancreatic cancer. Therefore, it is necessary to reconsider NAC regimens for pancreatic cancer.

## Introduction

Pancreatic carcinoma in Japan causes >28,000 deaths annually, with an overall 5-year survival rate of <5% (1,2). For patients with a localized disease, radical surgery may have long-term benefits. Routine treatment to improve prognosis in patients with carcinoma of the pancreatic head includes radical pancreatic resection comprising wide lymph node dissection and complete removal of the extra-pancreatic nerve plexus of the superior mesenteric artery (SMA) or the celiac axis (3–5). However, even in patients who undergo resection, 5-year survival is poor with a rate of 7–24%, and median survival is ∼1 year in most cases, indicating that surgery alone is inadequate. These unsatisfactory results are likely to be attributable to early vascular dissemination as metastases are present in most patients at the time of diagnosis (6). This hypothesis underpins the investigation of adjuvant chemotherapy following surgery. Oettle *et al* (7) reported that adjuvant chemotherapy with gemcitabine (GEM) produced a statistically significant improvement in survival.

A significant limitation of adjuvant therapy for pancreatic cancer is that 20–30% of the patients are ineligible for the designated therapy due to post-operative complications, such as delayed surgical recovery, patient refusal, comorbidity or early disease recurrence (8–10). This may be overcome by the use of neoadjuvant therapy in order that more patients receive potentially beneficial treatment. Other theoretical advantages of this approach include: early treatment of micrometastases; delay in surgery, thereby sparing those who already have occult metastases the morbidity and mortality associated with major surgery when disseminated disease becomes apparent at the time of reassessment; reduced risk of tumor seeding at the time of surgery; improved tolerance compared with post-operative therapy and a reduction in overall treatment time.

Potential disadvantages of neoadjuvant therapy include: a requirement for biliary decompression prior to chemotherapy and the potential for complications associated with biliary stents; delayed surgery, allowing progression to a non-resectable stage in patients whose disease does not respond to therapy; a lack of pre-operative tissue diagnosis (due to a risk of seeding when pre-operative biopsy is performed) and the potential for an increase in post-operative complications.

GEM is a deoxycytidine analogue that competes for incorporation into DNA to inhibit DNA synthesis. GEM is currently the standard treatment for advanced pancreatic cancer on the basis of a randomized study in 126 patients comparing GEM with 5-fluorouracil (5-FU), which confirmed a small but clinically important survival advantage and improved clinical response for GEM (11).

S-1 is an oral fluorinated pyrimidine developed by Taiho Pharmaceutical Co., Ltd. (Tokyo, Japan). The agent contains tegafur (FT), 5-chloro-2,4-dihydroxypyridine (CDHP) and potassium oxonate (Oxo) at a molar ratio of 1:0.4:1, and is based on a biochemical modification of 5-FU (12). FT, a pro-drug of 5-FU, is gradually converted to 5-FU and is rapidly catabolized by dihydropyrimidine dehydrogenase (DPD) in the liver. CDHP is a competitive inhibitor of 5-FU catabolism, and is ∼180 times more potent compared with uracil in inhibiting DPD (13). When FT is combined with CDHP, the resulting high 5-FU levels are maintained in the plasma and the tumor tissue. Additionally, CDHP has been suggested to have the potential to enhance the *in vivo* antitumor activity of 5-FU against subcutaneous tumors in nude mice, using human pancreatic carcinoma cells with a high tumoral DPD activity (14). Oxo inhibits the enzyme orotate phosphoribosyltransferase, the major enzyme responsible for 5-FU activation in colon cancer (15). Oxo preferentially localizes in the gut rather than the tumor, thereby selectively inhibiting the formation of 5-FU nucleotides in the gut and theoretically reducing gastrointestinal side-effects (16). The administration of oral S-1 is more convenient and simulates the effect of continuous 5-FU infusion. The safety and usefulness of combination chemotherapy with GEM and S-1 for advanced pancreatic cancer have been recently reported (17–19), while a phase III (GEST) trial in Japanese patients showed the non-inferiority of S-1 for GEM (20).

Furthermore, the usefulness of pre-operative GEM-based chemotherapy for the survival of patients with resectable pancreatic cancer has been recently reported (21–23). However, the combination regimen of GEM and S-1 for patients with pre-operative resectable pancreatic cancer has yet to be investigated. We previously conducted a pilot study of neoadjuvant chemotherapy (NAC) with the combination of GEM plus S-1 for resectable pancreatic cancer (24). Although NAC with GEM plus S-1 regimen is potentially effective for pancreatic head cancer, the optimal dosing strategy has not been determined. Therefore, the present phase I study on the treatment with combined GEM plus S-1 therapy in Japanese patients with pre-operative resectable pancreatic cancer was conducted to determine the maximum-tolerated dose (MTD) of each drug.

## Patients and methods

### Patient selection

Patients with radiologically proven resectable pancreatic cancer were included in this study. Additional inclusion criteria were age between 20 and 79 years, Eastern Cooperative Oncology Group (ECOG) performance status of ≤1 (ambulatory and capable of self-care), adequate renal function (normal serum creatinine and blood urea nitrogen levels), liver function [total bilirubin level, <2.5 times the upper normal limit (UNL) or <3 times the UNL after biliary drainage when the patient had jaundice and serum transaminase (GOT, GPT) levels, <2.5 times the UNL or <3 times the UNL after biliary drainage when the patient had jaundice], bone marrow reserve (white blood cell count, 4,000–12,000 mm^3^; neutrophil count, >2,000 mm^3^; platelet count, >100,000 mm^3^ and hemoglobin level, >9.5 g/dl) and pulmonary function (PaO_2_, >70 mmHg). Patients previously treated for cancer via tumor resection, as well as chemo-, immuno- or radiotherapy, were required to have discontinued treatment for at least 4 weeks prior to study enrollment.

Exclusion criteria were: pulmonary fibrosis or interstitial pneumonia, marked pleural or pericardial effusion or marked peripheral edema, severe heart disease, difficult-to-control diabetes mellitus, active infection, pregnant or lactating women, women of childbearing age unless using effective contraception, severe drug hypersensitivity, appearance of distant metastases during pre-operative chemotherapy, severe neurological impairment or mental disorder, active concomitant malignancy and other serious medical conditions.

Written informed consent was obtained from each patient prior to inclusion in the study. This study was approved by the Institutional Review Board of the Kanazawa University Hospital (Kanazawa, Japan).

### Study design

**Figure 1.**
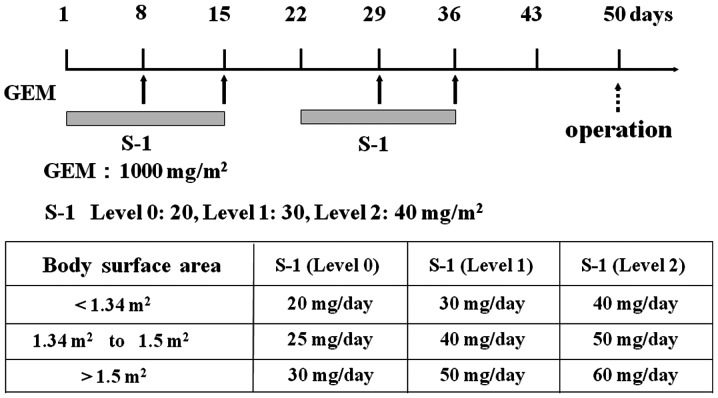
Treatment protocol for neoadjuvant chemotherapy (NAC) with gemcitabine (GEM) and S-1. S-1 (20–40 mg/day) was administered orally for 14 consecutive days, and GEM (1,000 mg/m^2^) was administered on days 8 and 15. In the NAC group, surgery was performed >2 weeks after 2 cycles of chemotherapy.

This was an open-label, single-centre, non-randomized, dose-escalation phase I study. The laboratory tests assessing eligibility were completed within 7 days prior to treatment initiation. S-1 was administered orally post-prandially for 14 consecutive days (from the evening of day 1 to the morning of day 15), followed by a 1-week break. Each capsule of S-1 contained 20 or 25 mg of FT. Individual doses were rounded down to the nearest pill size less than the calculated dose, given the available formulation. GEM was administered as a 30-min intravenous infusion on days 8 and 15 of each cycle. The cycle was repeated twice every 21 days pre-operatively. Surgery was performed >14 days after the termination of chemotherapy. This schedule was based on an *in vitro* study, which showed maximum synergy when fluoropyrimidine preceded exposure to GEM (25). The dose of each drug in this study was: level 0, S-1 20 mg/m^2^/day and GEM 1,000 mg/m^2^; level 1, S-1 30 mg/m^2^/day and GEM 1,000 mg/m^2^; level 2, S-1 40 mg/m^2^/day and GEM 1,000 mg/m^2^ (Fig. 1).

### Definition of dose-limiting toxicity (DLT) and MTD

DLT was determined during the two treatment cycles. DLT was defined, using the National Cancer Institute Common Toxicity Criteria scale (version 4.0), as one or more of the following effects attributable to the study drug: i) grade 3/4 neutropenia complicated by fever; ii) grade 4 neutropenia lasting for >4 days; iii) grade 4 thrombocytopenia; iv) any other grade 3/4 non-hematologic toxicity, with the exception of anorexia, nausea and vomiting in the absence of an appropriate anti-emetic and v) delay of recovery from treatment-related toxicity for >2 weeks. At least 3 patients were enrolled at each dose level. When DLT was observed after the first cycle in >2 patients, dose treatment was discontinued. When DLT was observed after the first cycle in 1 patient, 3 additional patients were placed on that dose level. When only 1/6 patients experienced DLT, then dose escalation was continued. There was no dose escalation in individual patients. The MTD of the combination was defined as the dose level that produced DLT in >2/6 patients or in the 3 initial patients. The recommended dose (RD) was defined as the dose level that was one level below the MTD considering the toxicity and tolerability in an outpatient setting.

### Assessment of efficacy

Tumor responses were evaluated according to the Response Evaluation Criteria in Solid Tumors (RECIST). Complete response (CR) was defined as the disappearance of the clinical evidence of the measurable tumor. Partial response (PR) was defined as a ≥30% reduction in the sum of the products of two perpendicular diameters of the measurable lesions compared to the baseline values, with no evidence of new lesions. Stable disease (SD) was defined as <30% reduction or <20% increase in the sum of the products of two perpendicular diameters of the measurable lesions compared to the baseline values, with no evidence of new lesions. Progressive disease (PD) was defined as an increase of ≥20% in the sum of the products of two perpendicular diameters of the measurable lesions compared to the baseline values, the appearance of any new lesion, or deterioration in the clinical status consistent with disease progression. To assess objective responses, patients were evaluated after 2 cycles of pre-operative chemotherapy.

### Pathological diagnosis

The surgically resected specimens were immediately fixed in 10% neutral-buffered formaldehyde solution. The specimens were cut horizontally into 5-mm tissue blocks (26), dehydrated and embedded in paraffin. Sections (5-*μ*m) were then cut and stained with hematoxylin and eosin (H&E). Each section was carefully examined using light microscopy. The tumors were evaluated according to the General Rules for the Clinical and Pathological Study of Pancreatic Cancer proposed by the Japanese Pancreatic Cancer Group. The grading system of Evans *et al* (27) was used to assess the pathological effects of pre-operative chemotherapy. The degree of cytological changes and tumor destruction were graded on a scale of I–IV: grade I, presence of characteristics of cytologic changes of malignancy, but little (<10%) or no evident tumor cell destruction; grade IIa, destruction of 10–50% of tumor cells; grade IIb, destruction of 51–90% of tumor cells; grade III, presence of few (<10%) viable tumor cells; grade IIIM, presence of sizable pools of mucin; grade IV, presence of no viable tumor cells; and grade IVM, presence of acellular pools of mucin.

## Results

### Clinical data of the patients

Between October, 2009 and May, 2012, 13 patients (6 men and 7 women) diagnosed with resectable pancreatic cancer were included in this study. Patient characteristics are provided in Table I. Treatment was initiated at level 1 in 3 patients. During the second course, adverse events were observed in 2 patients (grade 3 liver injury or neutropenia), leading to a dose reduction to level 0. Patient no. 1 was a 61-year-old woman with pancreatic body cancer. This patient was not administered GEM on day 15 during the first course due to grade 3 leukocytopenia. Patient no. 2 was a 65-year-old man with pancreatic body cancer. In this case, the first course of therapy was completed. However, the onset of the second course was delayed for >2 weeks and the trial was discontinued, due to grade 3 neutropenia and grade 2 thrombocytopenia. Patient no. 3 was a 38-year-old man with pancreatic head cancer. In this case, the first course was completed. However, during the second course a 20% reduction of the S-1 dose was necessary due to grade 3 liver dysfunction. Since DLT was observed after the first cycle in 2/3 patients, the level 1 trial was terminated and the dose level was switched to level 0.

At level 0, 10 cases were registered, while 2 patients were excluded. One case was complicated by portal vein thrombosis following percutaneous transhepatic cholangiodrainage for obstructive jaundice, and in 1 patient, post-operative pathological examination demonstrated an absence of invasive ductal adenocarcinoma. Therefore, only 8 cases were included in the analyses.

### Toxicity and post-operative complications

The most common toxicities observed during the 2 cycles of chemotherapy are provided in Table II. Of the 8 patients at level 0, 3 patients were not administered GEM on day 15 during the first course due to adverse effects: 1 patient had grade 3 liver dysfunction, 2 had grade 3 and 1 had grade 4 neutropenia, and the third patient had grade 3 anorexia. In these patients, the second cycle of chemotherapy was administered without S-1. Thus, at level 0, DLT was observed in 3/8 patients who reached MTD.

Surgery was performed on all the 11 patients included in this study. Post-operative complications included pancreatic fistulas in 5/11 patients at levels 1 and 0 [2 of grade A and 3 of grade B, according to the International Study Group on Pancreatic Fistula (ISGPF) classification (28)] and sepsis with *Pseudomonas aeruginosa* in 1 patient who underwent pancreaticoduodenectomy with SMA and superior mesenteric vein resection and reconstruction.

### Efficacy

Two of the 11 patients (18%) exhibited PR and 9 (82%) SD. The value of CA19-9 prior to treatment was elevated (>37 IU/ml) in 3/11 patients, and it decreased in 2/3 patients. The value of DUPAN-2 prior to treatment was elevated (>25 IU/ml) in 4/11 patients, and it decreased in all 4 patients. Positron emission tomography and computed tomography (PET-CT) using ^18^F-fluorodeoxyglucose (FDG) was performed in 9/11 patients, prior and subsequent to pre-operative chemotherapy. In these patients, PET-CT showed FDG uptake corresponding to pancreatic tumor in 8 patients. In 6 patients, a decrease in the FDG maximum standardized uptake value (SUV_max_) was documented. Eight of the 11 tumor specimens showed histopathological evidence of tumor cell injury, although none of the patients exhibited a pathological CR. The treatment effect, based on the grading system by Evans *et al* (27), was grade I in 3, grade IIa in 6 and grade IIb in 2 patients.

## Discussion

Curative surgical resection is the only means of curing pancreatic cancer. However, the majority of pancreatic cancer resections are reported to be R1 (29), and even after undergoing curative resection, patients with pancreatic cancer face a 50–80% local recurrence rate and a 25–50% chance of developing distant metastases (6). We have previously reported that for patients with localized pancreatic cancer, radical pancreatic resection, consisting of wide lymph node dissection and complete removal of the extra-pancreatic nerve plexus of the SMA or celiac axis, improves outcomes (3–5). However, the long-term results are not satisfactory due to the high frequency of distant metastasis. Considering the unsatisfactory outcomes obtained thus far, adjuvant chemo-therapy is required. In particular, pancreatic head cancer, partly due to surgical stress, requires a long post-operative recovery period prior to chemotherapy administration. Therefore, pre-operative chemotherapy may be expected to reduce the risk of distant metastasis.

Nakamura *et al* (17) conducted a phase II clinical trial of S-1 combined with GEM for metastatic pancreatic cancer. In that trial, S-1 was administered for 14 consecutive days prior to GEM. Moreover, Nakahira *et al* (30) reported that pre-treatment with S-1 enhances GEM effects in pancreatic cancer xenografts. The mechanism of these enhanced effects is considered to be 5-FU-mediated upregulation of the major mediator of cell uptake of GEM, the human equilibrative nucleoside transporter 1. In this study, we adopted the regimen of Nakamura et al (17), however, the S-1 dose was reduced due to the high incidence of side-effects with this pre-operative chemotherapy. In other words, S-1 was used only as a biochemical modulator of GEM.

We have previously reported that NAC with GEM plus S-1 was well-tolerated and may be effective, particularly against pancreatic head cancer (24). This phase I study was considered to determine the appropriate doses of anticancer drugs. It has been proven that the therapeutic effect of GEM for pancreatic cancer is superior to 5-FU (11) and that GEM is the only drug recognized as being globally effective against pancreatic cancer. The fact that S-1 is non-inferior to GEM was demonstrated in the GEST trial (20), however, an additive effect of S-1 for GEM was not observed. Therefore, in this regimen, the dose of the GEM does not need to be reduced for the addition of S-1. This dose of S-1 is ineffective as monotherapy, but the completion rate of chemotherapy was reduced due to adverse events. This is consistent with the experimental result, according to which S-1 is able to potentiate the effects of GEM (28). However, in the present study, administration of 20 mg/m^2^/day (level 0) did not reflect RD. Therefore, it is necessary to re-examine the therapy regimen of combined GEM with S-1 for patients with pre-operative resectable pancreatic cancer. It remains to be determined whether GEM or S-1 can be used as NAC.

Numerous preoperative chemotherapy or chemoradiotherapies for pancreatic cancer have been reported. A number of promising phase II trials have reported excellent results, however, no phase III trials have been available until recently. Currently, the first phase III randomized controlled trial of pre-operative chemotherapy using GEM plus oxaliplatin for resectable pancreatic head cancer (NEOPAC study) is being conducted (31). However, in the present NAC study, *Pseudomonas aeruginosa* infection and an increase in grade B pancreatic fistulas were observed as post-operative complications. These results suggest that pre-operative chemotherapy increases the possibility of infection. Consequently, there is now a focus on synbiotic treatment for surgical patients following pre-operative chemotherapy.

In conclusion, NAC with GEM plus S-1 was not well-tolerated in this study. However, pre-operative chemotherapy may be effective especially against pancreatic head cancer. Therefore, it is necessary to consider alternative chemotherapy regimens for pancreatic cancer.

## Figures and Tables

**Table I.** Patient characteristics.

Characteristics	Level 0 (n=10)	Level 1 (n=3)
Gender, n		
Male	4	2
Female	6	1
Age (years)		
Median	64.5	54.7
Range	54–74	38–65
Location, n		
Head of pancreas	7	1
Pancreas body and tail	3	2

**Table II.** Toxicities of preoperative chemotherapy.

Toxicity	Level 0, course 1, n (%)		
	Grade 2	Grade 3	Grade 4
Leucopenia	4 (40.0)	0 (0.0)	0 (0.0)
Neutropenia	2 (20.0)	2 (20.0)	1 (10.0)
Thrombocytopenia	1 (10.0)	2 (20.0)	0 (0.0)
Liver injury	1 (10.0)	1 (10.0)	0 (0.0)
Anorexia	0 (0.0)	1 (10.0)	0 (0.0)

## References

[1] Foundation for Promotion of Cancer Research (FPCR) Number of deaths by cancer site (2011). http://ganjoho.jp/data/public/statistics/backnumber/2012/files/fig01.pdf.

[2] Ishii H, Furuse J, Boku N (2010). Phase II study of gemcitabine chemotherapy alone for locally advanced pancreatic carcinoma: JCOG0506. Jpn J Clin Oncol.

[3] Nagakawa T, Kurachi M, Konishi K, Miyazaki I (1982). Translateral retroperitoneal approach in radical surgery for pancreatic carcinoma. Jpn J Surg.

[4] Nagakawa T, Nagamori M, Futakami F (1996). Results of extensive surgery for pancreatic carcinoma. Cancer.

[5] Miwa K, Ohta T, Shimizu K (2004). Augmented regional pancreaticoduodenectomy for pancreas head cancer: combined resection of pancreas head and superior mesenteric artery and vein.

[6] Evans DB, Abbruzzese JL, Willett CG, DeVita VT, Hellman S, Rosenberg SA (2001). Cancer of the pancreas. Cancer: Principles and Practice of Oncology.

[7] Oettle H, Post S, Neuhaus P (2007). Adjuvant chemotherapy with gemcitabine vs observation in patients undergoing curative-intent resection of pancreatic cancer: a randomized controlled trial. JAMA.

[8] Klinkenbijl JH, Jeekel J, Sahmoud T (1999). Adjuvant radiotherapy and 5-fluorouracil after curative resection of cancer of the pancreas and periampullary region: phase III trial of the EORTC Gastrointestinal Tract Cancer Cooperative Group. Ann Surg.

[9] Spitz FR, Abbruzzese JL, Lee JE (1997). Preoperative and postoperative chemoradiation strategies in patients treated with pancreaticoduodenectomy for adenocarcinoma of the pancreas. J Clin Oncol.

[10] Yeo CJ, Abrams RA, Grochow LB (1997). Pancreaticoduodenectomy for pancreatic adenocarcinoma: postoperative adjuvant chemoradiation improves survival. A prospective, single-institution experience. Ann Surg.

[11] Burris HA, Moore MJ, Andersen J (1997). Improvements in survival and clinical benefit with gemcitabine as first-line therapy for patients with advanced pancreatic cancer: a randomized trial. J Clin Oncol.

[12] Shirasaka T, Shimamoto Y, Ohshimo H, Yamaguchi M (1996). Development of a novel form of an oral 5-fluorouracil derivative (S-1) directed to the potentiation of the tumor selective cytotoxicity of 5-fluorouracil by two biochemical modulators. Anticancer Drugs.

[13] Tatsumi K, Fukushima M, Shirasaki T, Fujii S (1987). Inhibitory effects of pyrimidine, barbituric acid and pyrimidine derivatives on 5-fluorouracil degradation in rat liver extracts. Jpn J Cancer Res.

[14] Takechi T, Fujioka A, Matssushima E, Fukushima M (2002). Enhancement of the antitumor activity of 5-fluorouracil (5-FU) by inhibiting dihydropyrimidine dehydrogenase activity (DPD) using 5-chloro-2,4-dihydroxypyridine (CDHP) in human tumor cells. Eur J Cancer.

[15] Peters GJ, van Groeningen CJ, Laurensse EJ, Pinedo HM (1991). A compression of 5-fluorouracil metabolism in human colorectal cancer and colon mucosa. Cancer.

[16] Takechi T, Nakano K, Uchida J, Mita A (1997). Antitumor activity and low intestinal toxicity of S-1, a new formulation of oral tegafur, in experimental tumor models in rats. Cancer Chemother Pharmacol.

[17] Nakamura K, Yamaguchi T, Ishihara T (2005). Phase I trial of oral S-1 combined with gemcitabine in metastatic pancreatic cancer. Br J Cancer.

[18] Ueno H, Okusaka T, Ikeda M (2005). A Phase I study of combination chemotherapy with gemcitabine and oral S-1 for advanced pancreatic cancer. Oncology.

[19] Nakamura K, Yamaguchi T, Ishihara T (2006). Phase II trial of oral S-1 combined with gemcitabine in metastatic pancreatic cancer. Br J Cancer.

[20] Ioka K, Ikeda M, Ohkawa S (2011). Randomized phase III study of gemcitabine plus S-1 (GS) versus S-1 versus gemcitabine (GEM) in unresectable advanced pancreatic cancer (PC) in Japan and Taiwan: GEST study. J Clin Oncol.

[21] Vento P, Mustonen H, Joensuu T (2007). Impact of preoperative chemoradiotherapy on survival in patients with resectable pancreatic cancer. World J Gastroenterol.

[22] Palmer DH, Stocken DD, Hewitt H (2007). A Randomized phase 2 trial of neoadjuvant chemotherapy in resectable pancreatic cancer: gemcitabine alone versus gemcitabine combined with cisplatin. Ann Surg Oncol.

[23] Sato N, Kurashima K, Nagai H (2009). The effect of adjuvant and neoadjuvant chemo (radio) therapy on survival in 1,679 resected pancreatic carcinoma cases in Japan: report of the national survey in the 34th annual meeting of Japanese Society of Pancreatic Surgery. J Hepatobiliary Pancreat Surg.

[24] Tajima H, Ohta T, Kitagawa H (2012). Pilot study of neoadjuvant chemotherapy with gemcitabine and oral S-1 for resectable pancreatic cancer. Exp Therap Med.

[25] Rauchwerger DR, Firby PS, Hedley DW, Moore MJ (2000). Equilibrative-sensitive nucleoside transporter and its role in gemcitabine sensitivity. Cancer Res.

[26] Makino I, Kitagawa H, Ohta T (2008). Nerve plexus invasion in pancreatic cancer. Spread patterns on histopathologic and embryological analysis. Pancreas.

[27] Evans DB, Rich TA, Byrd DR (1992). Preoperative chemoradiation and pancreaticoduodenectomy for adenocarcinoma of the pancreas. Arch Surg.

[28] Bassi C, Dervenis C, Butturini G (2005). Postoperative pancreatic fistula: an International Study Group (ISGPF) definition. Surgery.

[29] Esposito I, Kleeff J, Bergmann F (2008). Most pancreatic cancer resections are R1 resections. Ann Surg Oncol.

[30] Nakahira S, Nakamori S, Tsujie M (2008). Pretratment with S-1, an oral derivative of 5-fluorouracil, enhances gemcitabine effect in pancreatic cancer xenografts. Anticancer Res.

[31] Heinrich S, Petalozzi B, Lesurtel M (2011). Adjuvant gemcitabine versus NEOadjuvant gemcitabine/oxaliplatin plus adjuvant gemcitabine in resectable pancreatic cancer: a randomized multicenter phase III study (NEOPAC study). BMC Cancer.

